# Ligand Binding and Crystal Structures of the Substrate-Binding Domain of the ABC Transporter OpuA

**DOI:** 10.1371/journal.pone.0010361

**Published:** 2010-04-29

**Authors:** Justina C. Wolters, Ronnie P-A. Berntsson, Nadia Gul, Akira Karasawa, Andy-Mark W. H. Thunnissen, Dirk-Jan Slotboom, Bert Poolman

**Affiliations:** 1 Biochemistry Department, Groningen Biomolecular Sciences and Biotechnology Institute, Netherlands Proteomics Centre, Zernike Institute for Advanced Materials, University of Groningen, Groningen, The Netherlands; 2 Biophysical Chemistry Department, Groningen Biomolecular Sciences and Biotechnology Institute, University of Groningen, Groningen, The Netherlands; Massachusetts Institute of Technology, United States of America

## Abstract

**Background:**

The ABC transporter OpuA from *Lactococcus lactis* transports glycine betaine upon activation by threshold values of ionic strength. In this study, the ligand binding characteristics of purified OpuA in a detergent-solubilized state and of its substrate-binding domain produced as soluble protein (OpuAC) was characterized.

**Principal Findings:**

The binding of glycine betaine to purified OpuA and OpuAC (K_D_ = 4–6 µM) did not show any salt dependence or cooperative effects, in contrast to the transport activity. OpuAC is highly specific for glycine betaine and the related proline betaine. Other compatible solutes like proline and carnitine bound with affinities that were 3 to 4 orders of magnitude lower. The low affinity substrates were not noticeably transported by membrane-reconstituted OpuA. OpuAC was crystallized in an open (1.9 Å) and closed-liganded (2.3 Å) conformation. The binding pocket is formed by three tryptophans (Trp-prism) coordinating the quaternary ammonium group of glycine betaine in the closed-liganded structure. Even though the binding site of OpuAC is identical to that of its *B. subtilis* homolog, the affinity for glycine betaine is 4-fold higher.

**Conclusions:**

Ionic strength did not affect substrate binding to OpuA, indicating that regulation of transport is not at the level of substrate binding, but rather at the level of translocation. The overlap between the crystal structures of OpuAC from *L.lactis* and *B.*subtilis, comprising the classical Trp-prism, show that the differences observed in the binding affinities originate from outside of the ligand binding site.

## Introduction

**Figure 1:**
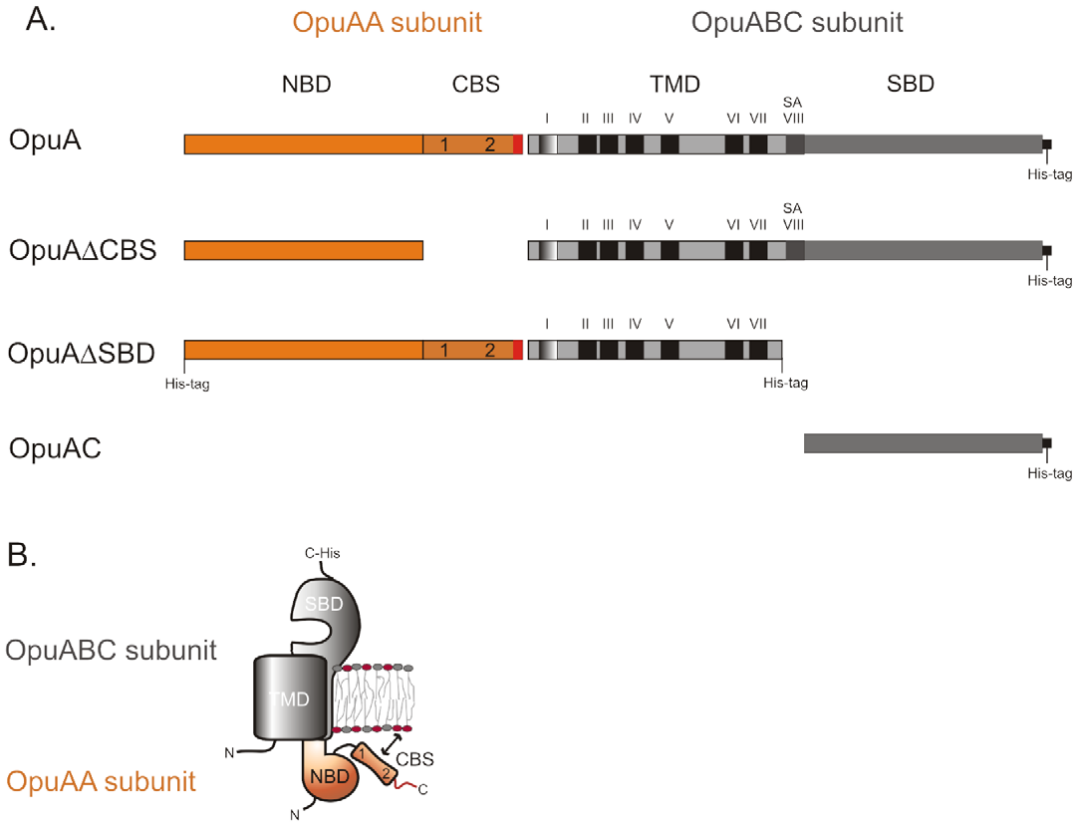
Schematic of OpuA from *L. lactis*. Panel A represents the different constructs used in this paper. OpuAA consists of the nucleotide-binding domain and tandem CBS domains connected to the anionic tail (approximately 20 amino acids, indicated in red). OpuABC consists of the transmembrane domain (TMD, OpuAB: I is amphipatic helix; II–VII are transmembrane segments; SA is the signal anchor sequence), connected to the substrate-binding domain (SBD, OpuAC). Panel B shows the organization of the subunits in the membrane. The functional protein is a dimer of the complex shown in panel B. The OpuABC subunits are depicted in grey, the OpuAA subunits in orange.

The osmoregulatory ABC transporter OpuA protects *Lactococcus lactis* against hyperosmotic stress by accumulating the compatible solute glycine betaine. It has been shown that osmotic activation of OpuA depends on three factors [1]–[3]: (i) the osmotic signal, associated with a change in the intracellular ionic strength; (ii) the membrane lipid composition, i.e. osmotic regulation requires threshold levels of anionic lipids; and (iii) the presence of tandem CBS domains (‘CBS module’) in OpuA that acts as osmosensor. Above threshold levels of anionic lipids and below the threshold ionic strength, the transporter is locked in an ‘off’ state, presumably via an interaction of the CBS module with the membrane surface. When the ionic strength is increased above the threshold or, alternatively, the negative surface charge of the membrane is decreased [1], [3], [4], the transporter is activated (‘on’ state). Because ionic strength and a negative surface charge act reciprocally, that is, the higher the fraction of anionic lipids - the higher the ionic strength needed for activation, it is thought that ions screen the electrostatic interaction of the CBS module with the membrane (as depicted in Fig. 1). We have recently shown that deletion of the CBS module (OpuAΔCBS mutant; Fig. 1A) or substitution of five surface-exposed cationic residues on the CBS module to neutral amino acids (OpuAK3R2 mutant) suffices for deregulated transport [5]. The OpuAΔCBS and OpuAK3R2 mutants are no longer osmotically regulated but otherwise fully functional in transport. The importance of the CBS module in osmoregulation has also been shown for an OpuA homolog in *Pseudomonas syringae* [6]. The mechanism of osmotic activation of OpuA bears resemblance to that of ProP from *Escherichia coli* [7]and BetP from *Corynebacterium glutamicum* [8], which are secondary transporters that use electrochemical ion gradients rather than ATP to drive transport. Whereas OpuA responds to ionic strength [2], BetP is specifically activated by K^+^ ions [9]. ProP also responds to ionic strength but additional factors such as hydration or macromolecular crowding and recently the membrane potential have been implicated in the osmotic regulation of transport [7], [10]. Studies on the transcriptional regulator of OpuA showed that the repressor of the *opuA* operon dissociates from the DNA at high ionic strengths, which is consistent with increased expression of the transporter and thus increased accumulation of glycine betaine at high osmotic stress [11].

The ABC transporter OpuA from *Lactococcus lactis* is a dimer and each half has two subunits. One subunit is composed of the nucleotide-binding domain (NBD) fused N-terminally to a tandem pair of CBS domains. The other subunit contains the transmembrane domain (TMD) fused N-terminally to the substrate-binding domain (SBD). The NBD-CBS and SBD-TMD subunits are named OpuAA and OpuABC, respectively (Fig. 1). In case of *L. lactis* OpuA, the NBD-CBS domains are followed by a stretch of about twenty amino acids, most of which are anionic. This anionic tail varies in length among OpuA homologues. It is lacking in the OpuA orthologue from *E. coli* (ProU), but can reach lengths of more than hundred amino acids long in some orthologues present in Archaea. For OpuA from *L. lactis* it has been shown that the anionic tail tunes the ionic regulation [2].

The SBD of OpuA, hereafter named OpuAC, belongs to a superfamily of proteins associated with ABC transporters involved in solute uptake in prokaryotes [12]. These proteins consist of two globular domains with a α/β fold that are connected by a flexible hinge. Relative movements of the domains about the hinge allow the proteins to adopt closed and open conformations. Substrates bind between the two domains and shift the equilibrium towards a closed state, a process often referred to as a Venus fy-trap mechanism [13]. The closed, ligand-bound proteins associate with the transmembrane domains and deliver the cargo for translocation. Previously, it was shown that OpuAC could complement OpuAΔSBD, albeit poorly due to the low affinity of OpuAC for the membrane-domain of OpuA [14]. OpuAC from *Bacillus subtilis* has previously been investigated in terms of ligand binding as well as having its structure determined [15], [16]. This protein is membrane-tethered via a N-terminal lipid modification rather than covalently linked to the translocator domain as in OpuA from *L. lactis*. We have now characterized ligand binding to the full-length transporter OpuA and to isolated OpuAC (see Fig. 1A). Furthermore, we have determined the crystal structures of OpuAC in the absence and presence of its natural ligand, glycine betaine, at a resolution of 1.9 Å and 2.3 Å, respectively.

## Results

### Glycine betaine binding

**Figure 2:**
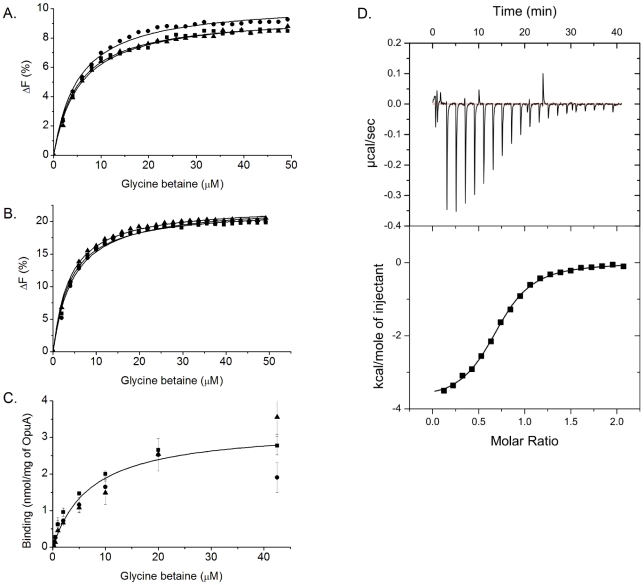
Glycine betaine-binding to purified OpuA (panel A & C) or OpuAC (panel B & D). Binding was measured using intrinsic protein fluorescence (panel A & B), filter-based assay using radio-labeled ligand (panel C), and isothermal titration calorimetry (panel D) at various concentrations of potassium phosphate (10–250 mM KP_i_, pH 7.0). The intrinsic protein fluorescence measurements and the filter-based assay were done using a concentration of ∼0.5 µM purified OpuAC. The fluorescent measurements were plotted as the absolute percentage change of the initial fluorescence signal in the absence of substrate (panel A & B); the filter-based measurements were corrected for background signals (panel C); and the data fits are shown as black lines. In panel A–C: measurements were done at 250 mM KP_i_ (▪), 50 mM KP_i_ (•) and 10 mM KP_i_ (panel A and C) or 12.5 mM KP_i_ (panel B) (▴). ITC measurements (panel D) were performed at 50 µM of purified OpuAC and the integrated heat peaks were fitted to a one site-binding model (black line, lower panel).

Binding of glycine betaine to purified OpuA (solubilized in DDM) and OpuAC was assessed under different conditions relevant for the osmoregulatory function of the transporter. Glycine betaine binding was measured with three different methods: (i) intrinsic protein fluorescence; (ii) isothermal titration calorimetry; and (iii) a filter-based assay using radioactive ligand. Measurements were performed at different ionic strengths, using increasing concentrations of potassium phosphate (ranging from 10–250 mM KP_i_, which is equivalent to an ionic strength of 0.02–0.5. The intrinsic protein fluorescence of OpuA and OpuAC decreased by the addition of glycine betaine until saturation was achieved. The maximal fluorescence change was about 9–11% for OpuA (Fig. 2A, Table 1) and 23–24% for OpuAC (Fig. 2B, Table 1). These differences reflect the larger number of tryptophan residues in OpuA (16 in total of which 10 are present in the OpuAC domain). The dissociation constants for glycine betaine binding to OpuA (K_d_'s around 5–6 µM, Fig. 2A, Table 1) and OpuAC (K_d_'s around 4–5 µM, Fig. 2B, Table 1) were similar and did not show any salt dependence. At very high protein concentration, the amount of glycine betaine bound was 0.74 mol/mol OpuAC (Figures S1). Although fluorescence measurements report binding constants over a wide concentration range, a K_D_ of ∼5 µM is generally too high for an accurate estimate of the number of binding sites from the intercept of the slope and the plateau of the binding curve [17]. We then determined the number of sites from the binding of radiolabeled glycine betaine to purified OpuA via ammonium-sulfate precipitation [18]. Again, the dissociation constant was shown to be independent of the ionic strength (Fig. 2C). The K_d_ was averaged from duplicate measurements for all salt concentrations, and was 7.3 µM; the maximal number of binding sites was ∼3 nmol/mg OpuA (which equals to 1.2 mol of substrate per mol OpuA or 0.6 mol per mol of substrate binding domain). To ascertain that glycine betaine binding only occurs to the OpuAC domain and not to some intracellular regulatory site, as has been observed for other ABC transporters [19], [20], ligand binding to OpuAΔSBD and OpuAΔCBS was also tested. Deletion of the CBS domains did not alter the dissociation constant and OpuAΔSBD did not show any glycine betaine binding (data not shown). In principle, ligand binding could occur to the membrane domain as suggested, for instance, by the crystal structures of full length transporters [21]. However, this binding would most likely be of low affnity and not readily detected. Our studies indicate that high affinity (K_D_ in the low µM range) only occurs to the extracytoplasmic substrate-binding domains of OpuA.

**Table 1:** Glycine betaine binding to purified OpuA and OpuAC using intrinsic protein fluorescence.

KPi, pH7.0 (mM)	OpuA		OpuAC	
	K_D_ (µM)	ΔF_max_ (%)	K_D_ (µM)	ΔF_max_ (%)
12.5	6.0±0.5	9.9±0.3	4.5±0.7	23.6±1.1
50	6.0±0.7	10.8±0.4	4.6±0.1	23.4±0.9
250	5.2±0.2	9.3±0.2	4.1±0.0	23.1±1.2

Dissociation constants (K_D_) and maximal fluorescence change (ΔF_max_) values of glycine betaine binding to OpuA and OpuAC at different salt concentrations. The dissociation constants and ΔF_max_ values were averaged from at least two independent measurements and the errors indicate the range.

### Thermodynamic analysis of ligand binding

Isothermal titration calorimetry (ITC) was used to determine the thermodynamic parameters contributing to the ligand binding. The heat changes upon ligand binding do not only yield information on the dissociation constant (and thus ΔG) and the number of binding sites, but also on the enthalpy (ΔH) and entropy (ΔS) changes. A representative measurement, corrected for blanks, is shown in Fig. 2D, which yielded a dissociation constant of 3.8±1.1 µM and 0.7 mol of glycine betaine bound per mol of OpuAC. The enthalpy change was −3.8±0.1 kcal mol^−1^ (exothermic effect) and TΔS was 3.6±0.1 kcal mol^−1^deg^−1^, yielding a Gibbs free energy of −7.5 kcal mole^−1^. Overall, the number of binding sites was less than unity (about 0.7 per mol of sites), which may reflect some endogenous substrate bound to a fraction of the purified protein (see below) and/or an overestimation of the protein concentration.

### Substrate specificity of OpuAC

**Figure 3:**
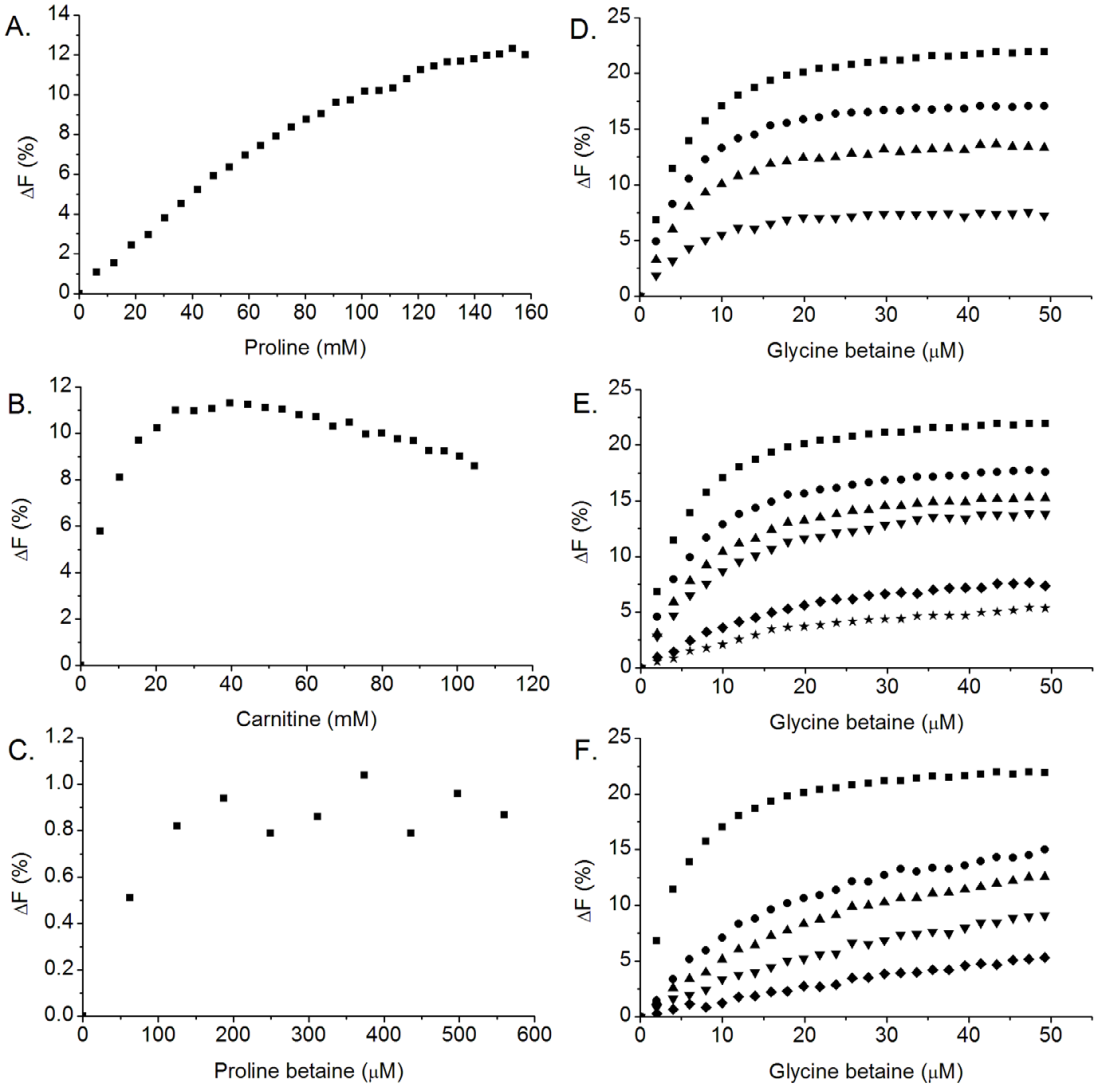
Substrate binding specificity of purified OpuAC. Binding was measured using intrinsic protein fluorescence at an OpuAC concentration of ∼0.5 µM. The following substrates were tested: proline (panel A), carnitine (panel B), proline betaine (panel C) and glycine betaine (Panel D–F). In the panels D–F, OpuAC was pre-incubated with different concentrations of proline (panel D), carnitine (panel E) or proline betaine (Panel F) as inhibitor, prior to titration with glycine betaine. In panel D–F, the concentrations of inhibitor are: no inhibitor (▪), 25 mM proline/carnitine or 0.125 mM proline betaine (•), 50 mM proline/carnitine or 0.25 mM proline betaine (▴), 100 mM proline/carnitine or 0.5 mM proline betaine (▾), 250 mM carnitine or 1 mM proline betaine (⧫) and 500 mM carnitine (★). Measurements in panel D–F were fitted with equation 1 (using a K_D_ of 4 µM for glycine betaine) and the inhibitor concentrations indicated. The inhibition experiments with proline and carnitine show a decrease in the ΔF_max_ at increased concentrations of inhibitor, which is caused by the high concentrations of inhibitor (see panel A and B). The fluorescence, however, reached the same end level in all measurements, which is indicative for specific and competitive binding.

Previously studied glycine betaine transport systems facilitate the accumulation of a wide variety of substrates [22], [23]. Binding of a number of these related substrates to OpuAC was determined by intrinsic protein fluorescence measurements as shown in Fig. 3. Proline betaine did not elicit significant fluorescence changes, but a K_I_ of 41±8 µM could be estimated from a (competitive) inhibition experiment by measuring glycine betaine-induced fluorescence changes. The affinity for proline was low and a dissociation constant of 132±20 mM was estimated. The binding curve for carnitine did not reach a stable end-level, and affinity constants were thus estimated as inhibition constants using glycine betaine as reporter (Fig. 3); the data are summarized in Table 2. Measurements in OpuA-containing proteoliposomes showed transport against a large concentration gradient of glycine betaine but not of carnitine (Figures S1). Proline slowly entered the proteoliposomes, but this effect was independent of the presence/absence of OpuA. In fact, calculations of the concentration of proline inside the proteoliposomes after 30 min (based on the assumption that the volume of the proteoliposomes is 1 µl/mg of lipid) showed no accumulation of the proline, indicating a diffusion-based process. Overall, the data indicate that OpuAC and thus OpuA is highly specific for glycine betaine and the related proline betaine with only a low affinity for other substrates.

**Table 2:** Binding of various ligands to purified OpuAC using intrinsic protein fluorescence.

Ligand	K_I_	K_D_
Proline betaine	41±8 µM	
Proline	198±55 mM	132±20 mM
Carnitine	65±11 mM	

Dissociation constants (K_D_) and inhibition constants (K_I_) of various ligands bound to OpuAC measured either directly (K_D_) or via inhibition of glycine betaine binding (K_I_), using intrinsic protein fluorescence. The dissociation constants were averaged from at least duplicate measurements, fitted independently to obtain the dissociation constants (for K_D_); for the K_I_-values, inhibition was measured over a range of inhibitor concentrations and the K_I_ values were averaged and the errors indicate the range.

### 3D Structure of OpuAC

**Figure 4:**
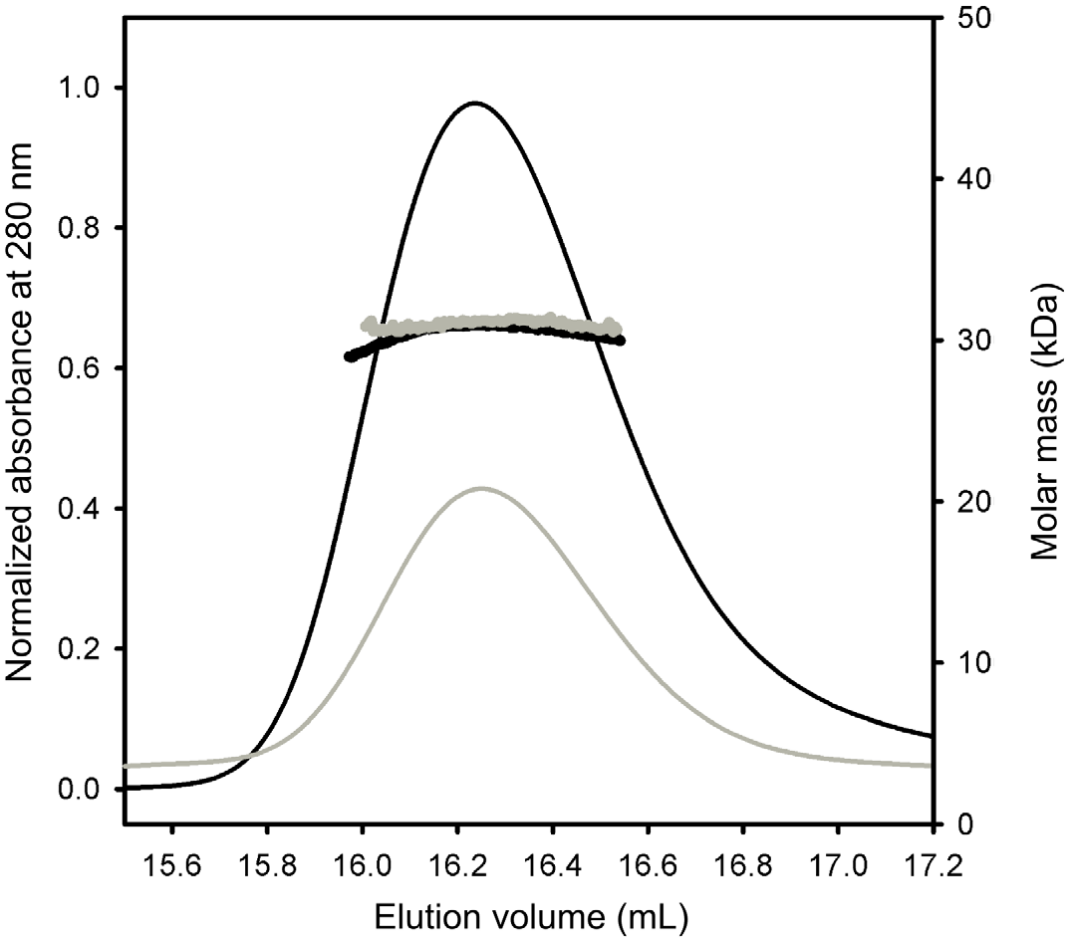
Static light-scattering analysis of purified OpuAC. Purified OpuAC, in the absence or presence of 1 mM glycine betaine, was run on a gel filtration column, coupled to detectors for UV absorbance, refractive index and light scattering. The molecular mass was calculated throughout the elution peaks.

The complete OpuA transporter consists of two OpuABC subunits, and thus contains two OpuAC domains. To investigate the possibility that the two substrate-binding domains interact, we determined the oligomeric state of isolated OpuAC by static light scattering coupled to size-exclusion chromatography (SEC-MALLS). OpuAC (Fig. 4) was found to have a molecular mass of 30.3 kDa, irrespective of the presence or absence of 1 mM glycine betaine, which matches a monomeric state of the protein, showing that the isolated domains do not form stable dimers or higher oligomers.

**Figure 5:**
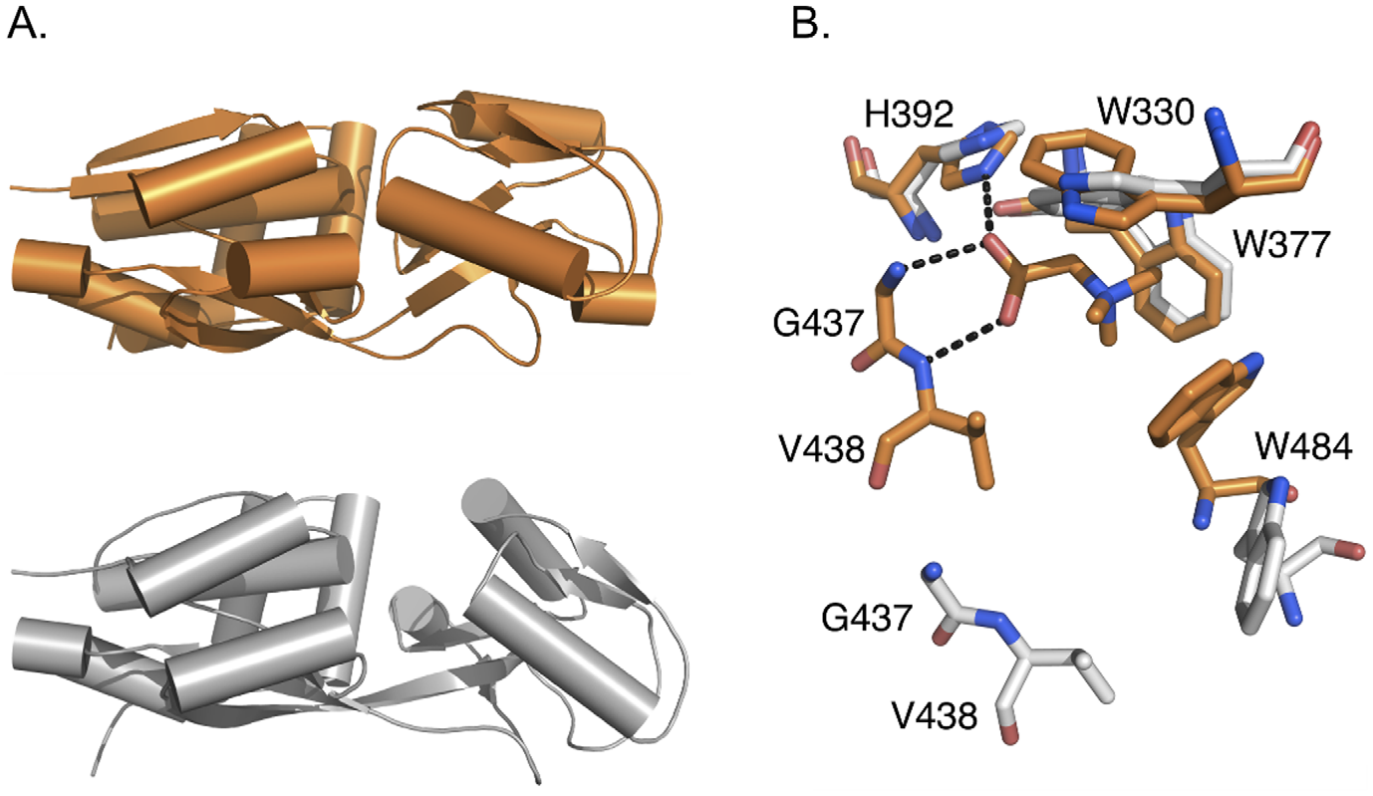
X-ray crystallography structure of OpuAC and its binding site. Panel A shows the structures of OpuAC in its closed (orange) and open (gray) conformations, highlighting the opening of the protein. Panel B, superimposition of the binding pockets for the open and closed-liganded structures of OpuAC. Upon closure of the protein a complete Trp-prism is formed for coordination of the quaternary ammonium moiety of glycine betaine. In addition, hydrogen bonds are formed between the carboxylate of glycine betaine and H392, G437 and V438, thereby stabilizing the closed conformation.

OpuAC was crystallized in the absence and presence of 1 mM glycine betaine, yielding an open conformation (space group P4_1_2_1_2, diffracting to 1.9 Å resolution) and closed-liganded conformation (space group H32, diffracting to 2.2 Å resolution), respectively (Table 3). The structures were solved by molecular replacement using the individual domains of the *B. subtilis* OpuAC structure as search models (Fig. 5).

**Table 3:** Data collection and refinement statistics.

	Open structure	Closed-liganded structure
**Data collection**		
Space group	P4_1_2_1_2	H32
Cell dimensions	a = b = 68.2 Å, c = 109.1 Å	a = b = 111.7 Å, c = 151.7 Å
Wavelength (Å)	0.872	0.872
Unique reflections	24,571	16,390
Completeness (%)	99.7 (98.9)	97.4 (96.1)
Resolution range (Å)	45-1.9	46-2.3
R_sym_	0.13 (0.45)	0.11 (0.54)
I/σ (I)	15.6 (3.9)	14.4 (4.5)
Average multiplicity	6.0 (6.0)	7.8 (7.3)
**Refinement**		
Resolution range	29-1.9	40-2.3
Number of reflections	24,526	15,646
R_work_/R_free_	0.17/0.21	0.16/0.20
No. atoms		
Protein	2064	2013
Water	285	140
Average *B*-factors (Å^2^)		
Protein	14.2	18.1
Water	21.6	25.7
R.m.s. deviations		
Bond lengths (Å)	0.005	0.008
Bond angles (°)	0.881	1.086
Ramachandran plot outliers (n, %)	1 (0.4%)	0 (0%)

The structures of OpuAC showed a fold typical of SBD's, having two α/β domains that enclose a ligand-binding site when in contact with each other (Fig. 5A). OpuAC belongs to Class II of the proteins in the SBD family [24], with two connecting segments forming the hinge, of which the central residues are Lys404 and Ser508. In the open conformation, the α/β domains had moved by 25° relative to one another when compared to the closed-liganded state. This falls in the normal range of opening of SBDs, which in typical class I and II SBDs range from 14° in the binding protein of the leucine transporter [25] to 60° in the binding protein of the leucine/isoleucine/valine transporter, both from *E.coli* [26]. During refinement, electron density accounting for bound ligand became visible in the ligand-binding site of the closed conformation structure. Glycine betaine in the closed-liganded conformation was tightly bound, with B-factors in the same range as for the rest of the protein. In the open conformation structure, electron density was observed in the binding site, although it could not be unambiguously assigned to glycine betaine.

### Ligand binding site

The superposition of the binding-site residues in the open and closed conformation is shown in Fig. 5B. In the closed-liganded conformation, the full binding site has been formed. Compared to the open state, a third tryptophan (W484) has moved in and contributes to the coordination of glycine betaine. The formed Trp-prism, previously described for OpuAC from *B. subtilis* [15], is well suited to coordinate the quaternary ammonium group of glycine betaine via cation-π interactions and van der Waals forces. In the closed-liganded conformation, the carboxylate group of glycine betaine is within optimal distance to form a hydrogen bond to H392, as well as to the backbone of G437 and V438; other residues that move in during the closure of the protein (Fig. 5B, orange).

## Discussion

The osmoregulatory ABC transporter OpuA accumulates glycine betaine in response to osmotic stress. Although ionic strength is the signal of the osmotic sensing mechanism, we now show that glycine betaine binding to the SBDs of OpuA is independent of the salt concentration in the medium. This was established in binding studies using the full size OpuA protein in a detergent-solubilized state as well as the isolated SBD domains expressed as soluble protein (OpuAC). Some glycine betaine transport systems have a rather broad substrate specificity, *e.g.* ProP from *E. coli*, which transports among others proline, glycine betaine and ectoine [23], and OpuC from *B. subtilis* which takes glycine betaine, crotonobetaine and carnitine together with more substrates [22]. Others, like BetP from *C. glutamicum*, are more specific to glycine betaine [27]. OpuA falls into the category of highly specific systems, as the dissociation constant for proline and carnitine is at least four orders of magnitude higher than that of glycine betaine.

### Structural basis for substrate-binding

The dissociation constant for glycine betaine binding (K_D_ ∼4 µM) to OpuAC (and thus OpuA) from *L. lactis* is about 4-fold lower than the corresponding value for the OpuAC homolog from *B. subtilis* (K_D_ ∼17 µM, [15]); similar differences are observed for proline betaine (K_D_ ∼41 µM and ∼295 µM, respectively [15]). At the level of primary sequence, OpuAC from *L. lactis* and *B. subtilis* are 44% identical. To find out what caused these differences, OpuAC was crystallized and its structure determined. Superimposing the Cα-backbone structures of OpuAC from *L. lactis* and *B. subtilis* yields an r.m.s.d of 0.98 Å. If one only superimposes the residues involved in the ligand binding, the r.m.s.d is 0.28 Å, implying that the binding sites are virtually identical (Figures S1). Since the binding sites do not differ, the explanation for the higher affinities in OpuAC from *L. lactis* must lie elsewhere in the protein. It has previously been shown for maltose-binding protein that mutating a residue which allosterically affects the equilibrium between the apo-state and the closed-liganded conformation can change the K_D_ for ligand binding by more than 2-orders of magnitude, while leaving the binding site intact [28]. The differences in substrate-binding activity between OpuAC from *L. lactis* and *B. subtilis* may have a similar basis and be caused by a lower flexibility in the *L. lactis* protein, which would imply a lower entropic barrier for domain closure and a decrease in *k_off_* (and thus K_d_) for the ligands.

Electron density was observed in the ligand-binding site of the open conformation structure. This density could not unambiguously be determined, but likely represents endogenous ligand that was co-purified with the protein, as ITC and fluorescence titration experiments showed that the amount of substrate bound per mol OpuAC was below 1. On the basis of modeling and refinement of the ambiguous density as glycine betaine, the initial coordination of the quaternary ammonium moiety would be performed by W377.

Several structures of proteins that bind quaternary ammonium compounds like glycine betaine have now been elucidated (e.g. BetP (PDB code 2WIT), AChBP (PDB code 1UV6), ChoX (PDB code 2REG), ProX from *E. coli* (PDB code 1R9L), and ProX from *A. fulgides* (PDB code 1SW2) [29]–[33]. ChoX from *S. meliloti* and ProX from *E. coli* are both SBPs with a similar fold as OpuAC, whereas BetP from *C. glutamicum* is a trimeric Na^+^-coupled symporter for glycine betaine. AChBP is a soluble homolog of the ligand-binding domain of nicotinic acetylcholine receptors, serving as a structural model for the pharmaceutically important family of pentameric ligand-gated ion channels [32], [34], [35]. Even though the overall structures of BetP, AChBP and OpuAC are unrelated, these proteins all share a common binding motif, with aromatic residues forming a box around the ligand and interacting via cation-π interactions. Likely this aromatic box is the optimal solution for the binding of the quaternary ammonium moiety. It is tempting to speculate that these systems have converged during evolution, very much like the independently evolved catalytic triad (Ser-His-Asp) in proteases [36].

Glycine betaine binding to OpuA is dependent on the environment of the protein. In a lipidic environment the dissociation constant is around 0.5 µM [14]), but in the DDM-solubilized state the dissociation constant is increased around 10-fold. It has previously been established that the lipidic environment is important for regulation of the OpuA transport activity [1]. Here we show that the membrane environment affects ligand binding as well but the effect is not related to the ionic sensing mechanism described previously [1].

### Conclusions

Substrate binding to OpuA was not affected by ionic strength, implying that ionic control of transport is at a later step. Also, the cooperativity observed in transport [14], [37] is not observed when binding of glycine betaine is probed. Overall, the data underline the importance of the lipidic membrane for the osmosensing and regulation of OpuA. The crystal structures of OpuAC show a classic Trp-prism binding pocket, forming the basis for the high affinity binding of glycine betaine, and reveal that the differences in ligand affinities between OpuAC from *L. lactis* and *B. subtilis* must lie outside of the ligand binding site.

## Materials and Methods

### Bacterial strains and growth conditions

Plasmids for expression of OpuA or derivatives were propagated in *Lactococcus lactis* strain NZ9000. The following OpuA mutants were used: OpuAΔCBS [1], and OpuAΔSBD, OpuAC [14]. The topologies of the different constructs are depicted schematically in Fig. 1A. The strains were cultivated semi-anaerobically in 2% (w/v) Gistex LS (Strik BV, Eemnes, NL), 65 mM potassium phosphate (KPi) pH 6.5, 1.0% (w/v) glucose and 5 µg/ml chloramphenicol at 30°C. For the isolation of the membrane vesicles, cells were grown in a 2 liter pH-regulated bioreactor to an OD_600_ of 2, after which transcription from the *nisA* promoter was initiated by the addition of 0.1% (v/v) culture supernatant from the nisin A producing strain NZ9700. The cells were harvested and stored at −80°C for preparation of membrane vesicles.

### Preparation membrane vesicles and protein purification

Cell disruption was performed by two passages through a cell disruptor (Constant Systems Ltd) at 39 kPsi in the presence of 1 mM MgSO_4_, 1 mM PMSF, 100 µg/mL DNase and 100 µg/mL RNase. The cell debris was removed from the solution by centrifugation (15 min 11,814×*g* at 4°C) and the membrane vesicles were collected by ultracentrifugation (60 min 185,000×*g* at 4°C). The pellet fraction (containing the membrane vesicles) was resuspended in 50 mM KP_i_, pH 7, 20% glycerol (Buffer A) to a concentration of ca. 20 mg/mL total protein. In case of preparation of the soluble OpuAC, the cell debris and membrane vesicles were spun down by ultracentrifugation in the presence of 200 mM KCl to prevent unspecific binding of OpuAC to the membrane vesicles.

#### Purification of OpuA

Membrane vesicles were collected by centrifugation (20 min 267,008×*g* at 4°C) and resuspended in buffer A plus 200 mM KCl to a final concentration of 5 mg/mL total protein. The membrane vesicles were solubilized in 0.5% DDM for 30 min on ice. The insoluble fraction was removed by ultracentrifugation (20 min 267,008×*g* at 4°C), after which the solubilized material was diluted five-fold in buffer A, supplemented with 200 mM KCl and 15 mM imidazole pH 8.0 to reduce the detergent concentration. The solubilisate was incubated with pre-equilibrated Nickel-Sepharose for 1–2 hours at 4°C under rotation, after which the resin (bed volume of 0.5 ml) was poured into a 10 mL column. The Nickel-Sepharose resin was washed with 10 mL of Buffer A containing 200 mM KCl, 15 mM imidazole and 0.05% DDM, after which the protein was eluted with Buffer A supplemented with 200 mM KCl, 200 mM imidazole and 0.05% DDM. OpuAΔCBS was purified using similar conditions, except that the imidazole concentration was increased to 50 mM imidazole for incubation and washing and 500 mM imidazole for elution of the protein.

#### Purification of OpuAC

The cell lysate from the disrupted cells after ultracentrifugation (supplemented with 50 mM imidazole) was mixed immediately with pre-equilibrated Nickel-Sepharose. Washing and elution was done in 50 mM KP_i_ pH 7, supplemented with 50 and 500 mM imidazole, respectively. OpuAC was purified further on a Superdex 200 10/300 Gl column in either 20 mM Na-MES pH 6.0, 150 mM NaCl (for crystallization purposes) or in 150 mM KP_i_ pH 7.0 (for biochemical characterization). Fractions containing OpuAC were pooled and concentrated, using a Vivaspin column with a cut-off of 10 kDa.

### Fluorescence measurements

Fluorescence spectra were obtained with a Fluorolog®-3 (Jobin Yvon) spectrofluorometer. A quartz-cuvette containing 800 µL of protein sample was incubated for 10 minutes at 25°C under constant stirring before stepwise addition of the substrate (or buffer as control), using a Hamilton syringe pump (Harvard apparatus). Samples were incubated for 5 seconds before the fluorescence signal was collected for 20 seconds to obtain an averaged value at each substrate concentration.

For fluorescence titration experiments, around 0.5 µM of purified OpuAC (in 150 mM KP_i_, pH 7) or OpuA (in 50mM KP_i_ pH 7, 20% glycerol, 0.05% DDM) was used, and a solution of glycine betaine was added in steps of 0.5 µL. For the intrinsic protein fluorescence measurements, the excitation and emission wavelengths were 295 and 360 nm (and slit widths of 1 and 5 nm), respectively. To determine the salt dependence of ligand-binding to purified OpuA, the buffer was exchanged to various KP_i_ concentrations (12.5–250 mM), pH 7.0, 20% glycerol and 0.05% DDM, using a NAP10 column (GE Healthcare). Corrections for background fluorescence changes were made by titrations with buffer.

### Data analysis

Fluorescence titrations were analyzed essentially as described by Lanfermeijer [17]. Curve fitting was performed in Origin (OriginLab). The dissociation constants for competitive binding were calculated using equation 1,
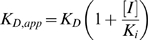
(1)in which K_D,app_ is the apparent K_D_ after fitting, K_D_ is the affinity constant for glycine betaine binding, [I] is the concentration of the competitive ligand, K_I_ corresponds to the K_D_ of the competitive ligand.

### Isothermal titration calorimetry (ITC)

Glycine betaine binding to OpuAC was measured by microcalorimetry on a ITC_200_ calorimeter (MicroCal) at 25°C. 200 µL of OpuAC (concentrated to 50 µM in 150 mM KP_i_, pH 7) was added to the cell. To determine the binding constant, glycine betaine (500 µM stock solution in the same buffer as the protein) was added stepwise. Typically, 20 injections of 2 µL volume were made with intervals of 120 seconds between each addition. The first titration in each experiment was 0.5 µL of glycine betaine instead of 2 µL, which was subsequently deleted in the data analysis; data were analyzed using the MicroCal software provided [38].

### Glycine betaine binding by filter-based assay (protein precipitation)

Binding of radio-labeled glycine betaine was also measured by the precipitation method [18]. This method is based on the principle that upon salting-out of the protein (by ammonium sulfate), the substrate remains trapped in the ligand-binding site. For these measurements, purified OpuA in the presence of 0.05% (w/v) *n*-dodecyl-β-maltopyranoside (DDM) was mixed such that the protein (at a final concentration of 100 µg/mL) was in a buffer composed of 10, 50 or 250 mM KP_i_ pH7.0, 20% glycerol and 0.05% DDM. The binding assays were carried in a volume of 100 µL. After 2 min of pre-incubation at 30°C, [^3^H]-glycine betaine was added to the assay mixture at the desired concentration (ranging from 0.1 µM to 42.5 µM) and the binding reaction was quenched after 2 min by dilution of the sample into 2 ml ice-cold 50% (w/v) ammonium sulfate solution. The mixture was filtered rapidly through 0.45 µm pore-size cellulose nitrate filters. The filters were washed twice with 2 ml ammonium sulfate solution. Subsequently, the filters were placed in an open plastic vial and dried under heating in an oven at 37°C for 2 hours. The radioactivity on the filters was measured via liquid scintillation counting, using emulsifier plus scintillation liquid (Perkin Elmer). Measurements were corrected for background signals and the average of the corrected data was fitted as described under fluorescence measurements. The specific activity of ^14^C-labeled glycine betaine was high enough for the transport assays (see below) but not for the ligand binding studies. We thus used ^3^H-glycine betaine in the binding studies. Radio-labeled [^3^H]-glycine betaine was prepared via a conversion of [^3^H]-choline chloride (Amersham, specific activity: 2.01*10^6^ MBq/mmol) to glycine betaine as described by Boch et al. [39].

### Substrate transport

The transport activity of OpuA was measured using [^14^C]-glycine betaine (converted from [^14^C]-choline chloride (Amersham, specific activity: 2.07*10^3^ MBq/mmol) as described by Boch et al. [39]), [^14^C]-L-proline (Amersham, specific activity: 8.58*10^3^ MBq/mmol) and [^14^C]-L-carnitine (GE Healthcare, specific activity: 2.11*10^3^ MBq/mmol) with OpuA reconstituted in proteoliposomes containing 38 mol% dioleoyl-phosphatidylglycerol, 50 mol% dioleoyl-phosphatidylethanolamine and 12 mol% dioleoyl-phosphatidylcholine. Proteoliposomes were prepared as described previously by Geertsma et al. [40] with an ATP regenerating system present and using 200 mM KP_i_, pH 7, as external medium. The transport activity was measured using a filter-based assay as described by Geertsma et al. [40] in a time dependent way (up to 30 min), using a fixed concentration of 50 µM or 5 mM of substrate.

### Static light scattering

An aliquot of 200 µl of OpuAC (0.2–0.4 mg/ml) was run at a flow rate of 0.5 ml/min on a Superdex 200 10/300GL gel filtration column (GE Healthcare) in 150 mM KP_i_ pH 7.0 (+/−1 mM GB) using an Agilent 1200 series isocratic pump at room temperature. Detectors were used for absorbance at 280nm (Agilent), static light-scattering (miniDawn TREOS Wyatt) and differential refractive index (Optilab Rex Wyatt). For data analysis, the ASTRA software package version 5.3.2.10 was used (Wyatt), with a value for the refractive index increment (dn/dc) protein of 0.187 ml/mg [41], [42].

### Crystallization and structure determination

OpuAC, in 20 mM Na-MES, pH 6.0 and 15 mM NaCl, was concentrated to 9 mg/mL. Crystals of OpuAC were grown by vapor diffusion in hanging drops. Crystallization conditions that yielded the open-liganded conformation consisted of 1 µL protein (9 mg/mL OpuAC) and 1 µL reservoir solution (0.2 M sodium iodide, 0.1 M Bis-Tris propane pH 8.5, 20% w/v PEG 3350). Crystals yielding the closed-liganded conformation were grown with 1 µL protein (9 mg/mL OpuAC plus 1 mM glycine betaine) and 1 µL reservoir solution (0.2 M NaCl, 0.1 M Na-Hepes, pH 7.0, 20% PEG 6000). Crystals of the open conformation were obtained after 21 days of incubation at 18°C, while for the closed-liganded conformation crystals were obtained after 4 days of incubation at 18°C. Crystals of the open and closed-liganded conformation were soaked in mother liquor supplemented with 12% glycerol for 30 s and 42% PEG 6000 for 30 s, respectively, and then fash cooled in liquid nitrogen. Data was collected to 1.9 Å and 2.3 Å resolution for the open and closed-liganded conformation, respectively, on beamline ID23-2 at the ESRF, Grenoble. Data processing, reduction and scaling were carried out using the program XDS [43]. The structures were solved by molecular replacement with the program Phaser [44], using the *B. subtilis* OpuAC structure (PDB code: 2b4l) as a search model. A few cycles of refinement using Refmac5 [45] and Phenix.refine [46], interspersed with manual model building using Coot [47], were necessary to complete the model. Water molecules were placed automatically in Fo-Fc Fourier difference maps at a 3 σ-cutoff level, and validated to ensure correct coordination geometries using Coot. Relevant statistics of the data collection and model refinement are given in Table 3.

### Accession codes

The coordinates have been deposited in the Proteins Data Bank with accession codes 3L6G and 3L6H.

## Supporting Information

Figures S1 Additional data: Figures S1, S2 and S3.
(0.83 MB DOC)
