# Supplementary material for: Ligand Binding and Crystal Structures of the Substrate-Binding Domain of the ABC Transporter OpuA
Source: PLoS One. 2010 Apr 29;5(4):e10361. doi: 10.1371/journal.pone.0010361 (PMC2861598; doi:10.1371/journal.pone.0010361)
Supplement: Figures S1 — Additional data: Figures S1, S2 and S3. (0.83 MB DOC) [file pone.0010361.s001.doc]

**Supplementary information**

**Figure S1, S2 and S3**

**Ligand binding and crystal structures of the substrate-binding domain of the ABC transporter OpuA**

Justina C. Wolters^1,*^, Ronnie P-A. Berntsson^1,*^, Nadia Gul^1^, Akira Karasawa^1^, Andy-Mark W. H. Thunnissen^2^, Dirk-Jan Slotboom^1^ and Bert Poolman^1^

^*^ These authors contributed equally to the work

^1^ Biochemistry Department or ^2^ Biophysical Chemistry Department

Groningen Biomolecular Sciences and Biotechnology Institute, Netherlands Proteomics Centre & Zernike Institute for Advanced Materials

University of Groningen

Nijenborgh 4, 9747 AG Groningen, The Netherlands

Correspondence to Bert Poolman

Tel: 0031 50 363 4190

Fax: 0031 50 3634165;

Email: [b.poolman@rug.nl](mailto:b.poolman@rug.nl)

**Figure S1. Determination of number of binding sites by intrinsic protein fluorescence**


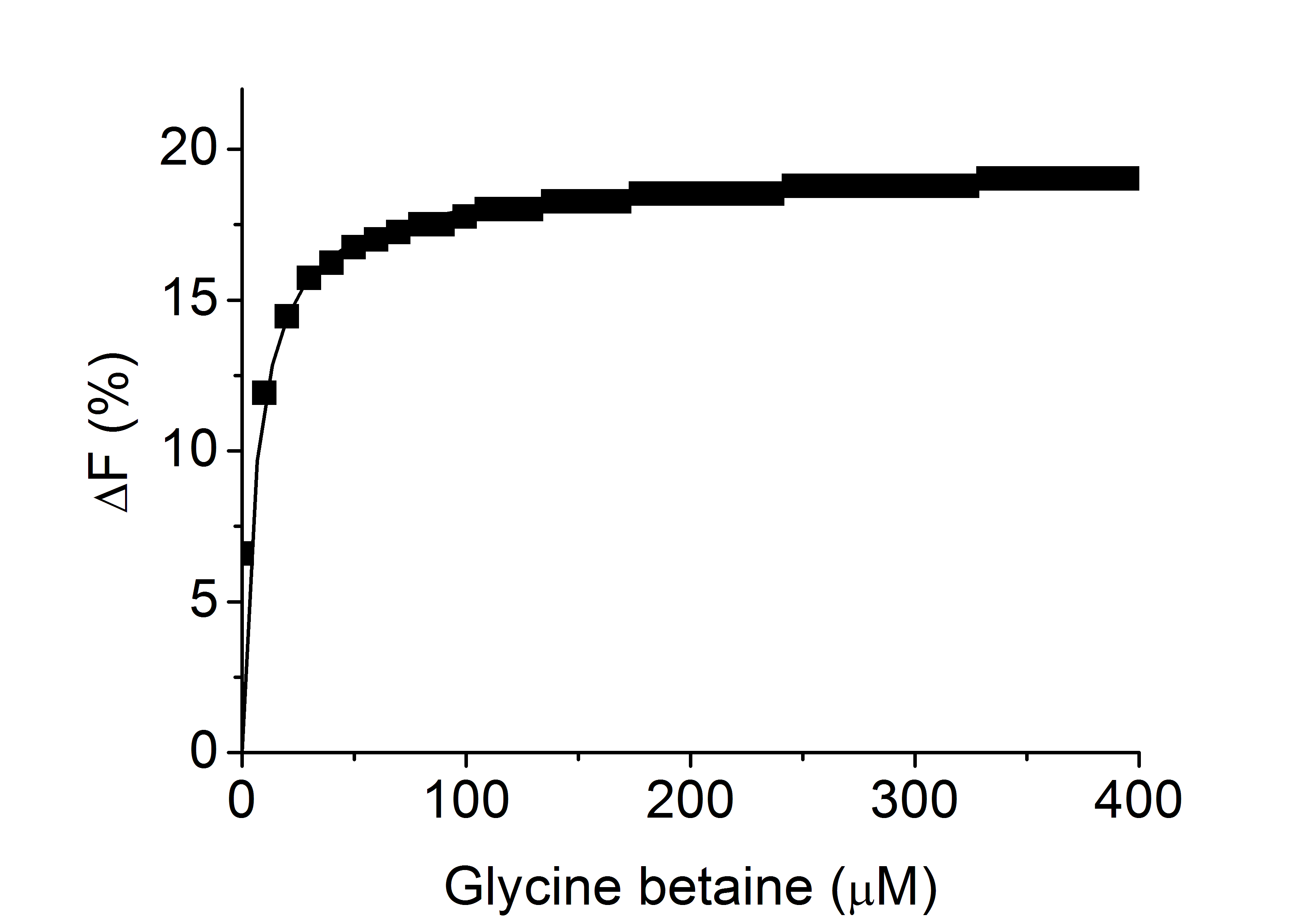


Figure S1. Glycine betaine binding to OpuAC measured by intrinsic protein fluorescence changes. 19.6 µM purified OpuAC in 150 mM KP_i_ pH 7.0 was titrated with increasing amounts of glycine betaine (steps of 10 µM glycine betaine). Glycine betaine titrations were corrected for dilution effects. The data fit (black line) yielded 14.5 ± 1.6 µM of bound substrate (which equals **0.74** mol glycine betaine per mol of OpuAC) and a K_D_ value of 8.0 ± 0.6 µM.

**Figure S2. Glycine betaine, proline and carnitine transport in proteoliposomes with or without purified OpuA**


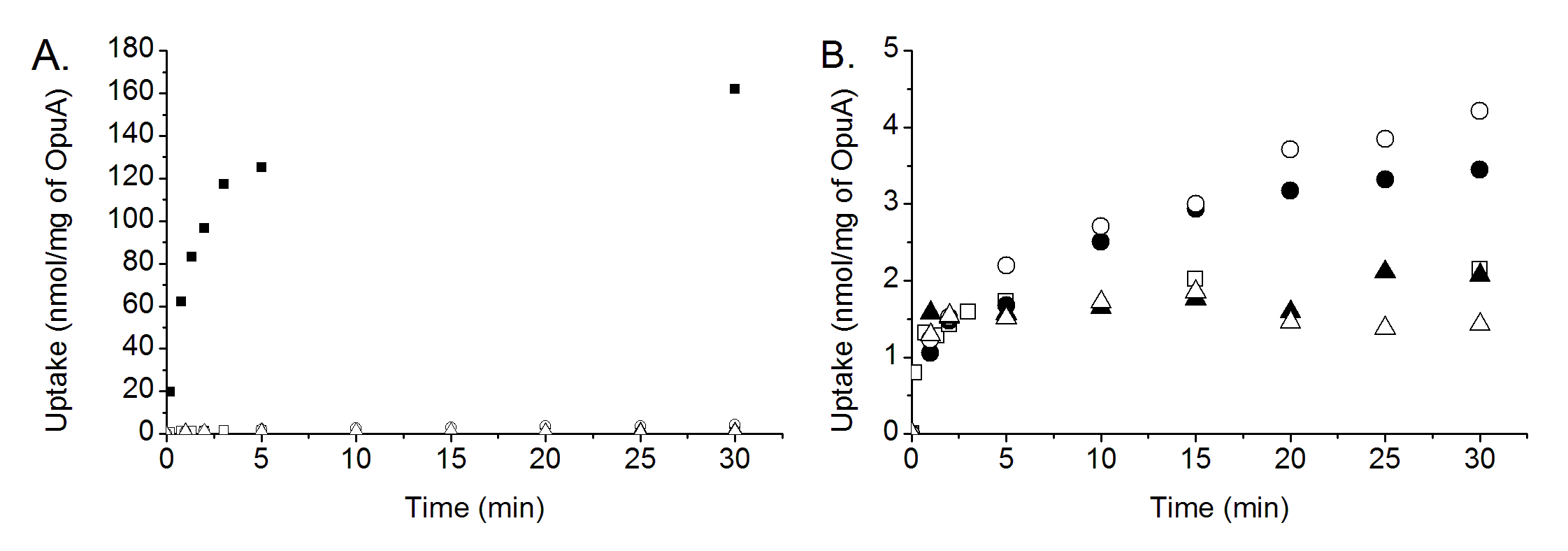


Figure S2. Transport activity of OpuA reconstituted in proteoliposomes composed of 38 mol% DOPG, 50 mol% DOPE and 12 mol% DOPC with the ATP-regeneration system enclosed inside the proteoliposomes. The following ^14^C-labeled substrates were tested: glycine betaine (◼), proline (⚫) and L-carnitine (▲). Transport was measured using a filter-based assay at a OpuA concentration of 60 µg/ml and with 51 µM (data shown) or 5 mM (not shown) of substrate. The data with 5 mM substrate are not shown but the results were similar as presented in the graphs with 51 µM: glycine betaine was transported against a concentration gradient; carnitine was not taken up, neither in the proteoliposmes nor in the empty liposomes; and proline entered the vesicles slowly but at an equal rate in the proteoliposomes and the liposomes devoid of OpuA. The data thus indicate that only the uptake of glycine betaine is facilitated by OpuA; proline and L-carnitine are bound by the SBDs of OpuA (see Fig. 3) but these substrates are not significantly transported.

**Figure S3. Overlay of *L.lactis* and *B.subtilis* binding sites**


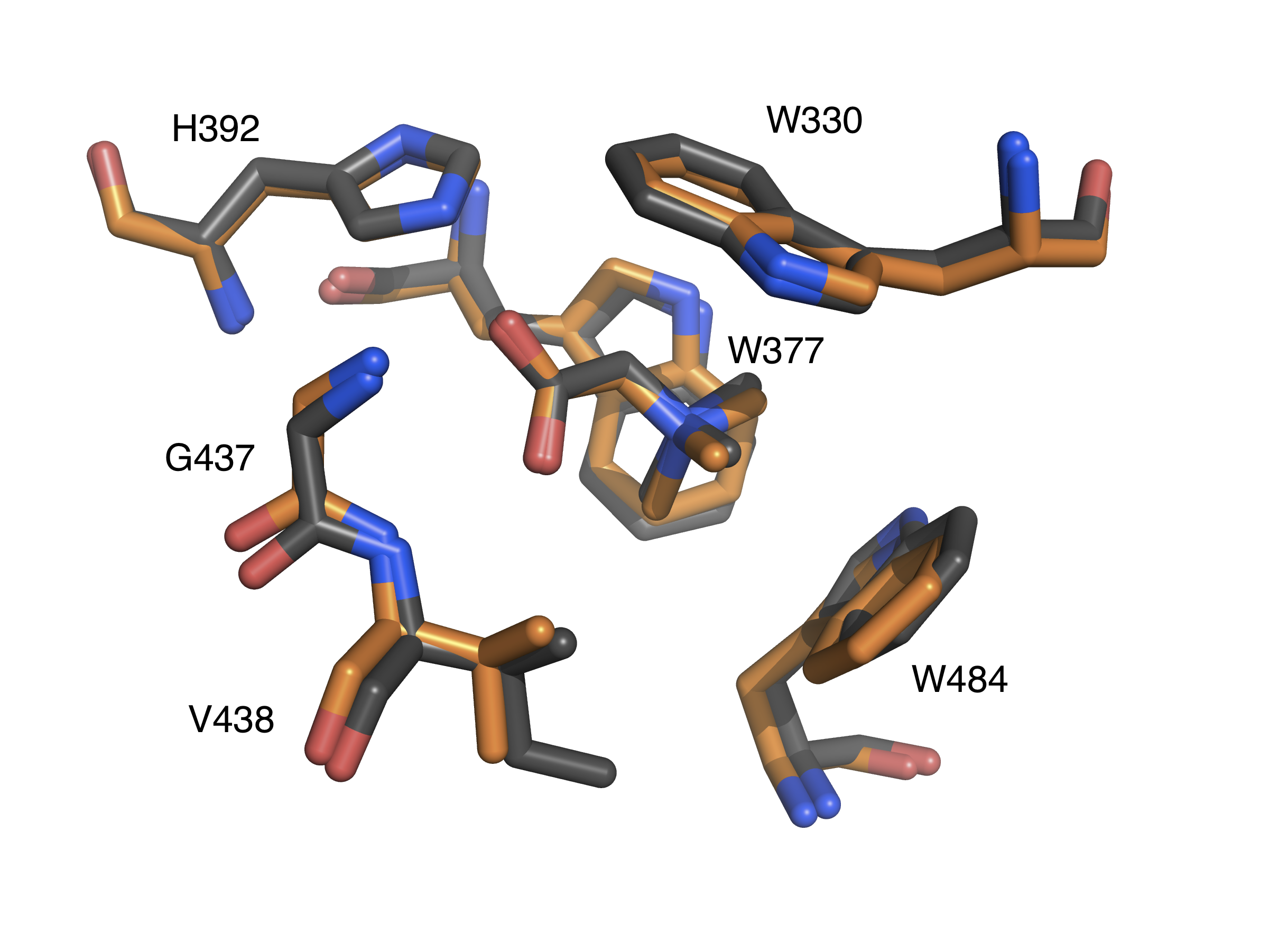


Figure S3. Overlay of the binding sites with bound glycine betaine of OpuAC from *L. lactis* (orange) and *B. subtilis* (grey). The residue numbering correspond to the residues in OpuAC from *L. lactis*. The r.m.s.d. of the superimposition was 0.28 Å.
